# AF9 sustains glycolysis in colorectal cancer via H3K9ac‐mediated PCK2 and FBP1 transcription

**DOI:** 10.1002/ctm2.1352

**Published:** 2023-08-10

**Authors:** Xuefeng He, Xinyang Zhong, Yi Fang, Zijuan Hu, Zhiyu Chen, Yaxian Wang, Huixia Huang, Senlin Zhao, Dawei Li, Ping Wei

**Affiliations:** ^1^ Department of Colorectal Surgery Fudan University Shanghai Cancer Center Shanghai China; ^2^ Department of Pathology Fudan University Shanghai Cancer Center Shanghai China; ^3^ Cancer Institute Fudan University Shanghai Cancer Center Shanghai China; ^4^ Institute of Pathology Fudan University Shanghai China; ^5^ Department of Oncology Shanghai Medical College Fudan University Shanghai China; ^6^ Department of Medical Oncology Fudan University Shanghai Cancer Center Shanghai China; ^7^ Emergency Department Shanghai Tenth People's Hospital Shanghai China

**Keywords:** AF9, colorectal cancer, glucogenesis, miR‐145

## Abstract

**Background:**

The tumourigenesis of various cancers is influenced by epigenetic deregulation. Among 591 epigenetic regulator factors (ERFs) examined, AF9 showed significant inhibition of malignancy in colorectal cancer (CRC) based on our wound healing assays. However, the precise role of AF9 in CRC remains to be explored.

**Methods:**

To investigate the function of AF9 in CRC, we utilised small interfering RNAs (siRNAs) to knock down the expression of 591 ERFs. Subsequently, we performed wound healing assays to evaluate cell proliferation and migration. In vitro and in vivo assays were conducted to elucidate the potential impact of AF9 in CRC. Clinical samples were analysed to assess the association between AF9 expression and CRC prognosis. Additionally, an Azoxymethane‐Dextran Sodium Sulfate (AOM/DSS) induced CRC AF9^IEC‐/‐^ mouse model was employed to confirm the role of AF9 in CRC. To identify the target gene of AF9, RNA‐seq and coimmunoprecipitation analyses were performed. Furthermore, bioinformatics prediction was applied to identify potential miRNAs that target AF9.

**Results:**

Among the 591 ERFs examined, AF9 exhibited downregulation in CRC and showed a positive correlation with prolonged survival in CRC patients. In vitro and in vivo assays proved that depletion of AF9 could promote cell proliferation, migration as well as glycolysis. Specifically, knockout of *MLLT3 (AF9)* in intestinal epithelial cells significantly increased tumour formation induced by AOM/DSS. We also identified miR‐145 could target 3′untranslated region of *AF9* to suppress AF9 expression. Loss of AF9 led to decreased expression of gluconeogenic genes, including phosphoenolpyruvate carboxykinase 2 (PCK2) and fructose 1,6‐bisphosphatase 1 (FBP1), subsequently promoting glucose consumption and tumourigenesis.

**Conclusions:**

AF9 is essential for the upregulation of PCK2 and FBP1, and the disruption of the miR‐145/AF9 axis may serve as a potential target for the development of CRC therapeutics.

## Graphical abstract

1. AF9 depletion facilitated colorectal cancer progression.

2. The miR‐145/AF9 axis holds promise as a potential therapeutic target.

3. AF9 modulated glycolysis by modifying the expression of phosphoenolpyruvate carboxykinase 2 (PCK2) and fructose 1,6‐bisphosphatase 1 (FBP1).

4. AF9 interacts with PCK2 and FBP1 through its recognition of H3K9ac.

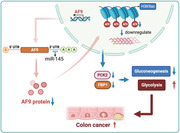

## 1 BACKGROUND

Colorectal cancer (CRC) is a common malignancy worldwide, ranking third in incidence and second in mortality among cancers.^1^ Metabolic reprogramming is a hallmark of CRC, with glycolysis being the predominant metabolic pathway providing energy and intermediates for tumour cell survival.^2^, ^3^, ^4^ Gluconeogenesis, the reverse process of glycolysis, represents a quiescent metabolic pathway in tumour cells.^5^, ^6^

The dysregulation of epigenetic processes, such as gene mutations and gene silencing, has been demonstrated to be critical in tumourigenesis and serves as potential targets for cancer therapies.^7^, ^8^ The identification of natural tumour suppressors is of paramount importance in counteracting uncontrolled tumour growth and inhibiting cancer progression. In the context of protein sequence alignment, a conserved domain called YEATS was discovered at the N terminus of and named after the five proteins: Yaf9, Eleven‐Nineteen Lysine‐rich Leukemia (ENL), ALL1‐fused gene from chromosome 9 (AF9), TATA‐binding protein‐associated factor 14 (Taf14) and Something about silencing protein 5 (Sas5).^9^ These YEATS domain‐containing proteins exhibit evolutionary conservation, with four identified in humans and three in *Saccharomyces cerevisiae*. These proteins are associated with various complexes involved in histone acetyltransferase activity, chromatin remodelling and transcription regulation.^10^ While the chromo (chromatin organisation modifier) domain and the plant homeodomain domain are known as histone lysine methylation ‘readers’, the bromodomain specifically recognises histone lysine acetylation18.^11^ Studies utilising modified histone peptide arrays and co‐crystallisation of YEATS‐acetylated H3 peptides have shown that the YEATS domain exhibits a high affinity for acetylated histone H3, specifically favouring H3K9 acetylation (H3K9ac).^12^ Notably, in childhood acute myeloid leukaemia, ENL utilises its YEATS domain to interact with acetylated histones, particularly H3K9ac and H3K27ac, thereby modulating the expression of oncogenic genes involved in the disease, whereas the involvement of *AF9* (also known as *MLLT3*) in solid tumour progression, including CRC, remains unknown.^13^

Fructose 1,6‐bisphosphatase 1 (FBP1) plays a critical role as an enzyme in the process of gluconeogenesis, where it catalyzes the conversion of FBP1 into fructose 6‐phosphate and inorganic phosphate. Importantly, FBP1 is well‐established as a tumour suppressor, exerting its suppressive effects on tumourigenesis. Intriguingly, aberrant DNA methylation has been observed to cause the loss of FBP1 expression in liver, colon, and gastric cancers.^14^, ^15^ It has been reported that FBP1 could target HIF1a and inhibits its transactivation independent of its enzymatic properties in kidney cancer.^16^ And our previous report revealed that FBP1 could be regulated by Forkhead Box C1 thus promoting CRC progression in CRC.^17^ Another significant enzyme in gluconeogenesis is phosphoenolpyruvate carboxykinase (PEPCK), which exists in two isoforms: the cytoplasmic form (PCK1) and the mitochondrial isoform (phosphoenolpyruvate carboxykinase 2 [PCK2]).^18^ PCK2 has been found to be overexpressed in various cancers, including lung, prostate, thyroid, bladder, breast, and cervix.^19^ However, in gluconeogenic tissues like the liver and melanoma, PCK2 acts as a tumour suppressor by facilitating significant cataplerosis to generate glucose, thus impeding glucose metabolism.^20^, ^21^ Additionally, methylation of the PCK2 promoter region has been linked to reduced PCK2 expression in renal cell carcinoma (RCC) and consequently inhibiting RCC progression. Therefore, these observations suggested a critical role of epigenetic regulation of FBP1 and PCK2 in modulating glucose metabolism in cancers. However, their downregulation in the regulation of CRC still needs further investigation. Discovering the mechanism of downregulating FBP1 and PCK2 could curb tumours and bring new hope for tumour treatments.

In this study, we discovered a novel tumour suppressor AF9, and its low expression could inhibit the expression of gluconeogenic genes, FBP1 and PCK2. Meanwhile, miR‐145 specifically targets the 3′ untranslated region (UTR) of AF9 mRNA to downregulate its expression. Therefore, disrupting the miR‐145/AF9 axis could be a potential strategy to maintain glucogenesis in normal tissues.

## 2 MATERIALS AND METHODS

Antibodies: Anti‐PCK2 (#6924) and anti‐FBP1 (#52804) antibodies (Cell Signaling Technology); Anti‐AF9 antibody (NBP2‐15303) and anti‐H3K18ac antibody (NBP2‐43535; Novus Biologicals); chromatin immunoprecipitation (ChIP) antibodies H3K9ac (07‐352) and H3K18ac (07‐354; Merck); β‐actin (66009‐1‐Ig; Proteintech).

Note: Antibodies were used at the following dilutions: 1:1000 for immunoblotting (IB), 5 μg per sample for IP or ChIP, and 1:100 or 1:200 for immunofluorescence (IF) or immunohistochemistry (IHC).

### 2.1 Cultivation of human CRC cell lines

The human CRC cell lines HEK293T, HT29, HCT116, DLD‐1, RKO, SW480, SW620, Ls174T and HCT8 were obtained from the American Type Culture Collection. Cells were cultured in Dulbecco's Modified Eagle Medium, Thermo supplemented with 2 mM glutamine, .1 mM nonessential amino acids, 1 mM sodium pyruvate and 10% fetal bovine serum (FBS). They were maintained at 5% CO_2_ at 37°C.

### 2.2 siRNA screening with wound healing assay

siRNA screen with wound healing assay was described as in a previous report.^22^ A comprehensive list of small interfering RNAs (siRNAs) targeting the 591 epigenetic regulatory genes was obtained from Dharmacon (Thermo) as described in a previous publication.^23^ HT‐29 cells of 2 × 10^4^ were seeded in 96‐well plates per well with black‐walled (Nunc) to grow for about 12 h. Then, transfections were performed using a robotic SX15 Handler workstation. The siRNAs transfection was performed using Lipofectamine RNAiMAX reagent (Thermo) according to the operation manual. Upon reaching 90% confluence post transfection, a stainless‐steel pin (Seiko) was used to create the scratch wounds, with a scratch of dimensions .75 × 4 mm. After scratching, the cells were gently washed with 1% FBS‐containing medium and grew for another 36 h. Real‐time monitoring of gap distances was measured with an IncuCyte high‐throughput screening system, allowing for continuous measurement and analysis of wound closure.

### 2.3 Plasmids, lentivirus package and infection

The cloning process involved the utilisation of HCT116 cDNA library for amplifying all open reading frames (ORFs) using KOD FX Neo DNA polymerase (KFX2, TOYOBO). The amplified ORFs were subsequently subcloned into different vectors. For AF9, it was subcloned into pCDH‐3′SFB vectors to generate FlAG‐AF9. Mutations in FlAG‐AF9 were introduced using the QuickChange site‐directed mutagenesis kit (#20051, Stratagene). The construction of FBP1 and PCK2 followed the same procedure as that of AF9. To generate shRNA‐resistant clones, Polymerase Chain Reaction (PCR) products containing four synonymous mutations in the middle of the shRNA target sequence were created. These PCR products were then subcloned into pCDH‐hygromycin vectors without any tags. The oligonucleotides for the construction of pGIPZ‐associated vectors can be seen in the Supporting Information Table.

### 2.4 Lentivirus package and infection

Standard procedures were employed for the amplification of lentivirus in subconfluent HEK293T cells. To infect CRC cell lines, polybrene (TR1003G, Merck) was added at a final concentration of 8 μg mL^−1^. The cells were incubated with the lentivirus mixture for 72 h. Subsequently, the cells were subjected to trypsin digestion and transferred to a fresh medium. The stable cell lines establishment was cultured from the cells sorted by their green fluorescence, with the desired expression or knockdown.

### 2.5 Cell proliferation and transwell migration assay

The cell proliferation assay was performed using the Cell Counting Kit‐8 (CCK‐8, CK04, Dojindo) according to the manual instruction. As for the transwell migration assay, the overexpression or knockdown cells were seeded in the upper chamber using the transwell filter inserts with 8 μm pores (#3422, Corning). After incubation for about 24 h, the number of cells that migrated to the lower surface of the chamber was calculated in five random fields (magnification, ×100). The experiments were conducted in duplicate and replicated three times to ensure reliable and consistent outcomes. The details of both assays can be referred to a previous report.^24^

### 2.6 Subcutaneous xenograft tumours

Animal experiments followed ethical guidelines provided by the Experimental Animal Center of Fudan University Shanghai Cancer Center (FUSCC). Female nude mice (BALB/c, SPF grade, 4−5 weeks old) were subcutaneously injected with 2 × 10^6^ cells per group. Tumour sizes were measured every 4 days using the formula: volume (mm^3^) = width^2^ (mm^2^) × length (mm)/2. After 8 weeks, mice were euthanised, and tumours were excised, fixed and sectioned for hematoxylin and eosin (H&E) staining and microscopic observations. The experimental procedures were approved by the Committee on the Ethics of FUSCC, following the Guide for the Care and Use of Laboratory Animals of the National Institutes of Health.

### 2.7 Transient transfection and quantitative real‐time PCR (qRT‐PCR)

For DNA transfection, a ratio of 1:3 (mg/mL) of DNA to Lipofectamine 3000 reagent (Invitrogen) was used. For siRNA and antigomiR transfection (Guangzhou Ruibo Biological Co. Ltd.), a ratio of 10:1 (nM/mL) of siRNA or antigomiR to RNAiMax was employed. The qRT‐PCR was carried out by total RNA extraction (NucleoZOL, Macherey‐Nagel), reverse transcription (ABScript II RT Mix, ABclonal), and real‐time quantification by using TB Green Premix Ex Taq II (TaKaRa). The quantification of miRNA by qRT‐PCR followed a previously reported method.^25^ The data were normalised to the reference gene β‐actin expression. The primer pairs can be found in the Supporting Information Table.

### 2.8 Western blotting

The cells were lysis by RIPA (Thermo) with protease inhibitor (Bimake), and the protein content was measured by Bicinchoninic Acid Assay kit, Thermo. Immunoblotting was applied to analyse the protein samples. Briefly, the protein samples were running on Sodium dodecyl sulfate‐polyacrylamide gel, Epizyme Biomedical Technology followed by transferring to .2 μm immobilon Polyvinylidene Fluoride membranes, Millipore Sigma. The acquired membranes with protein were then incubated with Quick blocking buffer (QuickBlock, Beyotime) for 30 min at room temperature, primary antibodies at 4°C overnight and finally the membranes were incubated with secondary antibodies at room temperature for about 1 h. Electrochemiluminescence (ECL) system (Share‐bio) was applied to detect the protein bands.

### 2.9 Collection of clinical CRC samples

This study was conducted in accordance with the approval of the Ethics Committee of FUSCC. Each patient participating in the study has received informed consent. The CRC samples were pathologically diagnosed and verified. Patients underwent standard chemotherapy after surgery. Tissue material usage complied with approved guidelines. CRC staging ranges from I to IV: Stages I–III represent nonmetastatic cancer, while stage IV indicates metastatic cancer, often involving the liver.

### 2.10 IHC analysis

The use of tissue samples for IHC analysis in this study was approved by the Ethics Committee of FFUSC. The tissue slices underwent deparaffinisation and rehydration using xylene and graded ethanol. Antigen retrieval involved autoclaving at pH 8.0 and 120°C for about 10 min. Endogenous peroxidase activity was blocked by incubating the slices in a solution of 3% H_2_O_2_ diluted in methanol. Primary antibodies were incubated overnight at 4°C, followed by the application of the secondary antibody at room temperature for 45 min. Staining with 3,3 ‐diaminobenzidine was performed for an appropriate duration, and counterstaining was done using Mayer's Hematoxylin solution. Magnification images of 20× were acquired, and positive cell counts were obtained from five random fields.

### 2.11 The construction of mouse model and IF staining

The *Villin‐cre* and *AF9* genetic mouse model used in this study was obtained as a gift from Prof. Wang's laboratory, and the detailed method can be found in their publication.^23^ The *AF9* ^IEC‐/−^ (*AF9* ^fl/fl^:*villin‐Cre*) mouse line was generated by breeding *AF9^fl/fl^* mice with villin‐Cre transgenic mice to delete the ninth exon. To confirm the recombination of *AF9^IEC‐/−^* (Figure 3C), a pair of primers was designed to detect the deleted allele as follows: Forward primer: 5′‐ATCCCTGTCCTTATCACCATCGCTT‐3′, reverse primer: 5′‐GAACTACAAAGCACAGCAATGAAGA‐3′. The length of the floxed *AF9* allele is 544 bp, and the deleted *AF9* allele is 411 bp.

Male mice aged 6−32 weeks were used for all animal studies. Mice were group‐housed, typically with 4−5 mice per cage, except for cases where less than 5% of mice required single housing due to the loss of cage mates. They were maintained under a 12‐h light/dark cycle and provided with ad libitum access to water and standard mouse chow. Humane care was provided to all mice throughout the study. All animal experiments were conducted in accordance with the guidelines and regulations approved by the Institutional Animal Care and Use Committee.

In this study, the impact of dietary administration on *AF9^IEC‐/^* mice and control wild‐type mice was assessed using a DSS‐induced inflammation model. On Day 1, the mice were given 2% DSS (w/v) in their drinking water for a duration of 5 days, followed by a period of 8 days with regular drinking water only. This DSS‐to‐water cycle was repeated a total of three times. On Day 36, all mice were euthanised, and a thorough necropsy was conducted. H&E staining or Zonula Occluden‐1 (ZO‐1) staining of paraffin‐embedded tissue sections was performed for the histopathological examination.

Following the instruction of standard protocols, the fixed tissues were stained with primary antibodies and then incubated with secondary antibodies conjugated to Alexa Fluor dyes and DAPI. Cell imaging was captured by Leica TCS SP8 WLL confocal laser microscope.

### 2.12 Prediction of miRNA target

The miRNA database (www.microrna.org) was used to predict potential miRNAs that may regulate AF9 gene expression. This database utilises support vector machines and high‐throughput training data to predict numerous miRNA‐target interactions. Using this approach, we obtained a list of miRNAs with the potential to target AF9 mRNA. To prioritise miRNAs with strong binding potential, we selected those with prediction scores above .6.

### 2.13 RNA sequencing and KEGG analysis

Total RNA underwent RNA sequencing on an Illumina HiSeq 2500 system. BaseSpace Sequence Hub was applied to analyse the resulting sequencing data. The clusterProfiler was used to perform Kyoto Encyclopedia of Genes and Genomes (KEGG) pathway analysis, and the generated plot was visualised by ggplot2 in R (version 4.3.1). The comparisons made were shAF9 versus shNT in the HT29 cell line and AF9 overexpression versus control (vector) in the HCT116 cell line. The co‐changed genes in both cell lines were identified. The RNA‐seq data have been uploaded, and the accession number for the SRA data is PRJNA944549.

### 2.14 Dual‐luciferase report assay

Dual‐luciferase activity was measured in HT29 and HCT116 cell lines by introducing the promoter of AF9 of tumour and normal tissue into pGL3B plasmid into above cells, according to the Operation Manual (Promega, E1910).

### 2.15 Measurement of metabolites, extracellular acidification rate assay (ECAR) and patients standardised uptake value (SUV)max

Cells with genetic manipulation were harvested, and intracellular metabolites were extracted using an aqueous solution of 80% (v/v) methanol. The extracted samples were subjected to metabolomic analysis using gas chromatography mass spectrometry (GC‐MS). The loading of samples and analysis of glycolytic metabolites from GC‐MS were performed following the protocols described in a previous study.^26^

Additionally, a total of 40 patients who underwent fluoro2‐D‐deoxyglucose F18 (18F‐FDG) Positron emission tomography (PET)/computed tomography (CT) examinations at FUSCC between 2019 and 2020 were included in the study. The imaging diagnoses were independently reviewed and analysed by two radiologists.

### 2.16 AF9‐Y78A cell line construction

To endogenously mutate homo sapiens *AF9* Y78 to A78, we used the following primers, including sgRNA and donor ssDNA, which were transiently transfected into the indicated CRC cells.

sgRNA: 5′‐CTTACAAAGTAGAAGAATCT‐3′; Donor ssDNA: 5′‐TTTTGTCTATTATTCTCATCTTACCTATTTTCTTTTTTAATGTTGCAGTGTGCAAAGATCCACCATATAAGGTTGAAGAATCTGGAGCTGCTGGTTTCATTTTGCCAATTGAAGTTTATTTTAAAAACAAGGTATGTAATCTTTACCCATTAATCTTTCA‐3′. The picked monoclonal cells were expanded in 48 well‐plate, and genomic DNA was extracted for sequencing to ascertain the mutation of Y78A in indicated CRC cells.

### 2.17 Co‐immuno‐precipitation assay

The cell lysate from FLAG‐AF9 cells was incubated with FLAG mouse monoclonal antibody‐coated beads (Sigma) for 4 h. The purified FLAG‐AF9 protein and complexes were then denatured by boiling at 95°C for 8 min. The samples were loaded onto an SDS‐PAGE gel to separate FLAG‐AF9 and its associated proteins. The separated proteins were detected by western blot using specific antibodies as indicated.

### 2.18 ChIP assay

For the ChIP assays, mouse monoclonal anti‐FLAG antibody (Sigma) at a dilution of 1:2000 and rabbit monoclonal anti‐H3K9ac antibody (Millipore) at a dilution of 1:500 was used. As a negative control, rabbit monoclonal IgG antibody (Cell Signaling) at a dilution of 1:500 was included. The presence of binding regions targeted by the specific antibodies was estimated by PCR. Parts of the DNA were reserved as an input control before antibody addition to serve as a normalisation reference for the ChIP assay. The PCR primer sequences that target DNA fragments within the promoters of interest were listed in Supporting Information Table. The experimental groups were normalised relative to their respective controls, of which the signals were deemed as 1.00.

### 2.19 Clinical samples

A total of 282 patients diagnosed with colorectal adenocarcinoma at FUSCC between 2008 and 2009, without prior preoperative therapy, were enrolled in this study. The patients were followed up for a median duration of 81 months, with a maximum follow‐up of 97 months. Informed consent was obtained from all participants, and the study was approved by the institutional review board of Shanghai Cancer Center.

### 2.20 Statistical analysis

Statistical data analysis was performed using appropriate tests based on the distribution of the variables. Unpaired Student's *t‐*tests were used for normally distributed variables when comparing two groups, while Mann–Whitney U tests were used for non‐normally distributed variables. For comparisons involving more than two groups, one‐way Analysis of Variance and Kruskal–Wallis tests were used as parametric and nonparametric methods, respectively. The linear relationship between two variables was determined using Pearson's or Spearman's correlation analysis. R software (version 4.3.1) and SPSS software (version 26.0) were used for all statistical analyses. Experimental data were typically presented as mean ± standard deviation, and three repetitions were set up to ensure the robustness of the results and conclusions. The level of statistical significance is indicated in the respective figure legends.

## 3 RESULTS

### 3.1 siRNA screening of epigenetic regulator factors (ERFs) discovers AF9 plays a suppressive role of CRC

**FIGURE 1:**
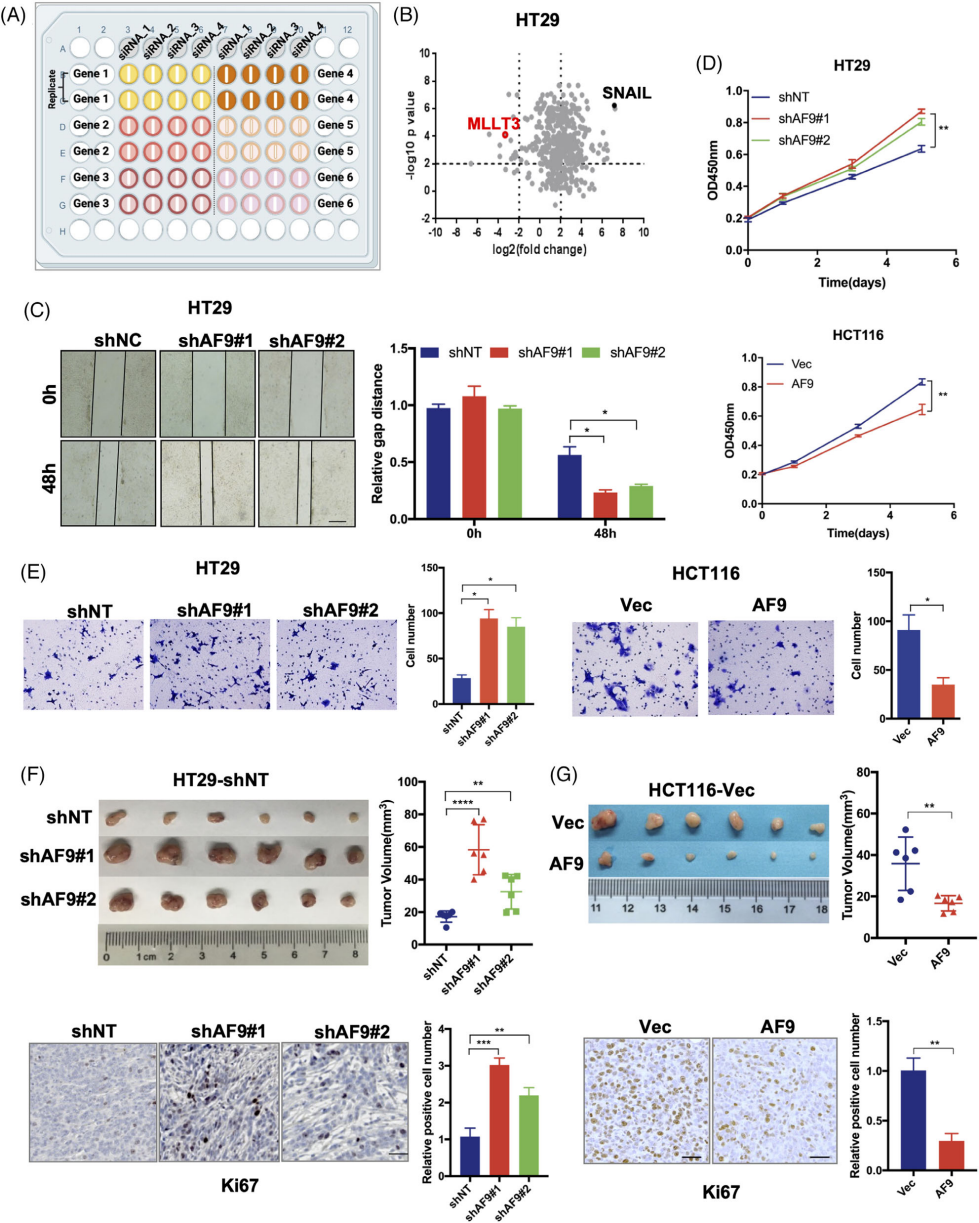
siRNA screening of epigenetic regulator factors (ERFs) discovers AF9 plays a suppressive role of colorectal cancer (CRC). (A and B) HT29 cells were transfected with individual siRNA targeting 591 ERFs (four siRNA for each gene) or siRNA targeting SNAI1 as the positive control or non‐targeting control siRNA (siNC) as the negative control, respectively. Scratch wound healing assays were performed. Real‐time gap distances were measured using IncuCyte high‐throughput screening system for 48 h. Schematic diagram of screening strategy was presented (A). Data presented as volcano plot of two independent experiments (B, top panel). The small interfering RNAs (siRNAs) with *p* value < .01 and migration distance (normalised to siNC) < .25 or > 4 was considered to be effective siRNAs that significantly influence tumour cell activities. ERFs targeted by more than two effective siRNAs were selected as candidate ERFs required for tumour cell activities (B). (C) Representative images of wound healing assays performed in HT29 cells with or without shAF9 were shown at indicated time point (scale bar represents 400 μm). (D) Cell proliferation of HT29 cells with or without shAF9, or HCT116 cells with or without AF9 overexpression was measured by CCK‐8. (E) Representative images of transwell assays performed in HT29 cells with or without shAF9 or in HCT116 cells with or without AF9 overexpression were shown (scale bar represents 60 μm). (F and G) Xenograft formation. HT29 cells with or without AF9 or HCT116 cells with or without AF9 overexpression were implanted into left groin of nude mice (*n* = 6 for each group). The tumour volume was measured at the end of the experiment. Ki67 staining was performed to test the in vivo proliferation (*p < .05, **p < .01, ***p < .001, ****p < .0001).

Dysregulation of ERFs plays important roles in the progression of tumours.^27^ To identify which ERF contributes to suppress tumour cell proliferation or migration in CRC, we adopted siRNAs to knock down 591 genes encoding ERFs in HT29 cells, followed by wound healing assays to examine tumour cell migration using IncuCyte high‐throughput screening system (Figure 1A). Among those ERFs, the knock down of AF9 with four independent siRNAs in HT29 cells significantly promoted the capacity of wound healing. Zinc‐finger transcription factor protein (SNAIL) was used as a positive control in this screening system (Figure 1B). We successfully repeated wound healing assays for HT29 cells with or without AF9 depletion (Figure 1C). As wound healing assay needs time span up to 48 h, the impact of AF9 on tumour cells has two aspects: proliferation and migration. We separated these two aspects by using transwell assay and CCK‐8 assay. To select the suitable cell lines for depleting AF9 or forced expression of AF9, we tested AF9 expression in 8 CRC cell lines (Supporting Information Figure S1A,B). We depleted AF9 in HT29 and DLD‐1 cells while forcing expression of AF9 in HCTT16 and RKO cells (Supporting Information Figure S1C). In Figure 1D, depletion of AF9 promoted cell migration while forced expression of AF9 impaired cell migration (Figures 1D and S1D). Meanwhile, CCK‐8 assay showed that depletion of AF9 in HT29 cells promoted cell proliferation while forced expression of AF9 in HCTT16 impaired cell proliferation (Figures 1E and S1E). The depletion of AF9 in HT29 cells significantly drives cell cycle progression after releasing for 24 h from cell synchronisation, while overexpression of AF9 in HCT116 cells induced cell cycle arrest after releasing 16 h from cell synchronisation, further verifying the role of AF9 in dampening cell proliferation (Supporting Information Figure S1G). We then performed a subcutaneous xenograft implantation experiment to assess the suppressive role of AF9 for CRC cells in vivo. Results showed HT29 cells with AF9 knockdown enlarged the volumes of solid tumour, compared to control groups, and Ki67 staining identified the enhanced tumourigenic capacity of HT29 cells without AF9 (Figure 1F). Reversely, a decreased tumour size was found when HCT116 cells with forced expression of AF9 and Ki67 staining showed a consistent result (Figure 1G). Therefore, AF9 plays a suppressive role in the xenograft formation of human CRC cells.

### 3.2 AF9 expression negatively associates with CRC progression

**FIGURE 2:**
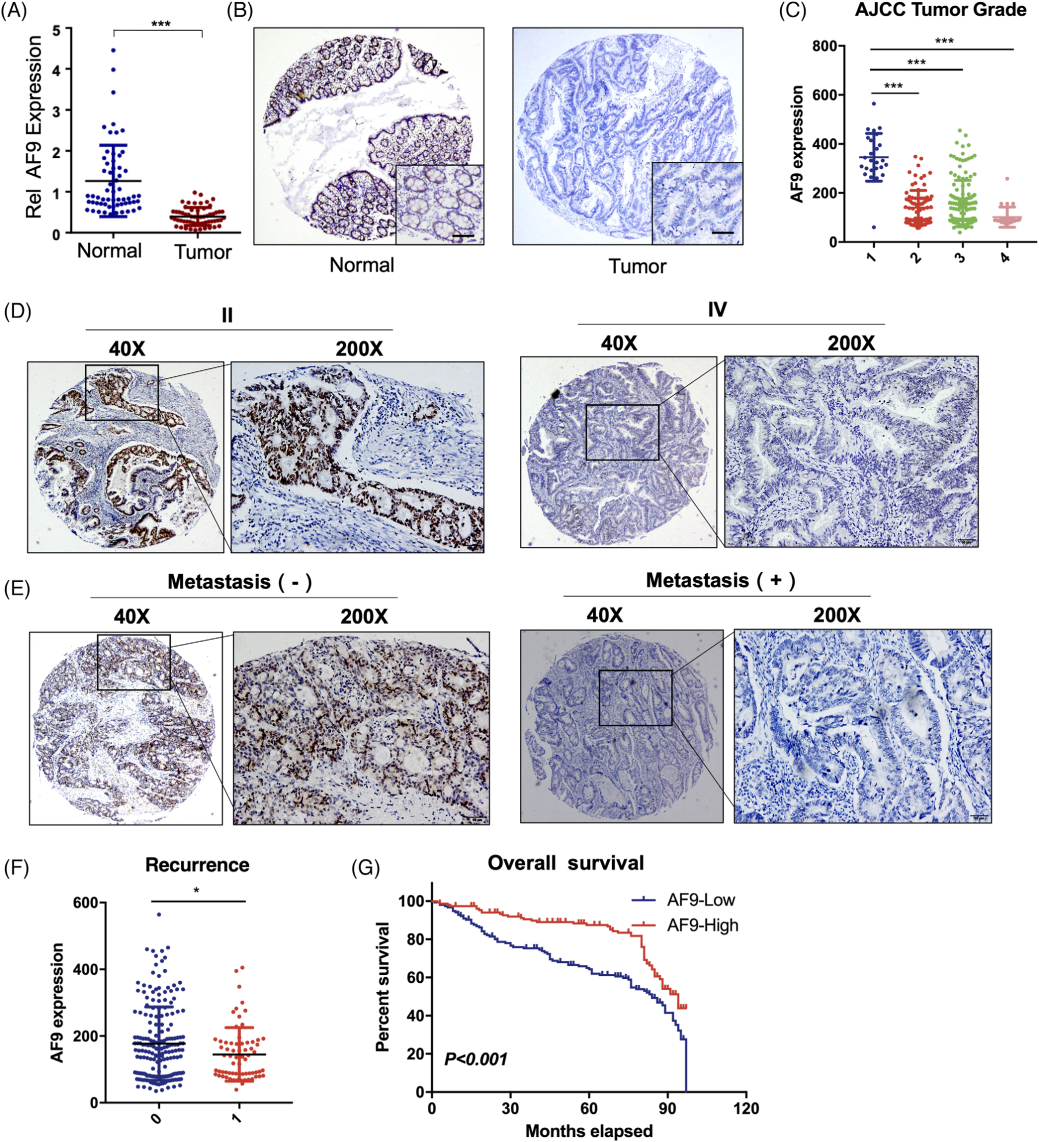
AF9 expression negatively associates with CRC progression. (A) AF9 mRNA levels in tumour tissues and adjacent tissues. (B) Representative images of immunohistochemistry (IHC) using antibody against AF9 in tumour tissue and paired adjacent tissues. (C) AF9 mRNA levels in tissues from patients with different stages of CRC. (D and E) Representative images of IHC using antibody against AF9 in tissues from different stages of CRC. (F) AF9 mRNA levels in tissues from patients with or without recurrence. (G) Survival curve. CRC patients in our hospital were divided into two groups according to AF9 expression levels in CRC tissues by IHC (*p < .05, ***p < .001).

To understand whether AF9 expression associates with CRC progression, we first tested AF9 expression using qRT‐PCR in 60 paired CRC samples. Results showed AF9 expression in tumours was dramatically downregulated, compared to adjacent normal tissues (Figure 2A). We identified AF9 protein level and distribution in the tumour and adjacent tissue and consistently, in tumour, AF9 was decreased. Otherwise, AF9 was high expression in normal tissue and localised in nuclear (Figure 2B). Further, we examine the association between AF9 expression and CRC progression. As shown in Figure 2C–E, as the CRC grade rises, AF9 expression is gradually lost or silenced, indicating AF9 could be used as a biomarker for monitoring CRC progression (Figure 2C,E). The recurrence of CRC is the major risky factor in patients’ survival duration. So, we examined AF9 expression in CRC primary samples with or without recurrence. In samples of patients without relapse, the expression level of AF9 is significantly higher than that of patients with relapse (Figure 2F). And the survival duration of CRC patients with high or low expression of AF9 also showed a negative correlation between AF9 expression and the prognosis of patients (Figure 2G). Hence, in CRC, the reduction of AF9 expression could be used as a progressive marker of CRC.

### 3.3 AF9 suppresses cancer initiation and progression in AOM/DSS‐induced CRC model

**FIGURE 3:**
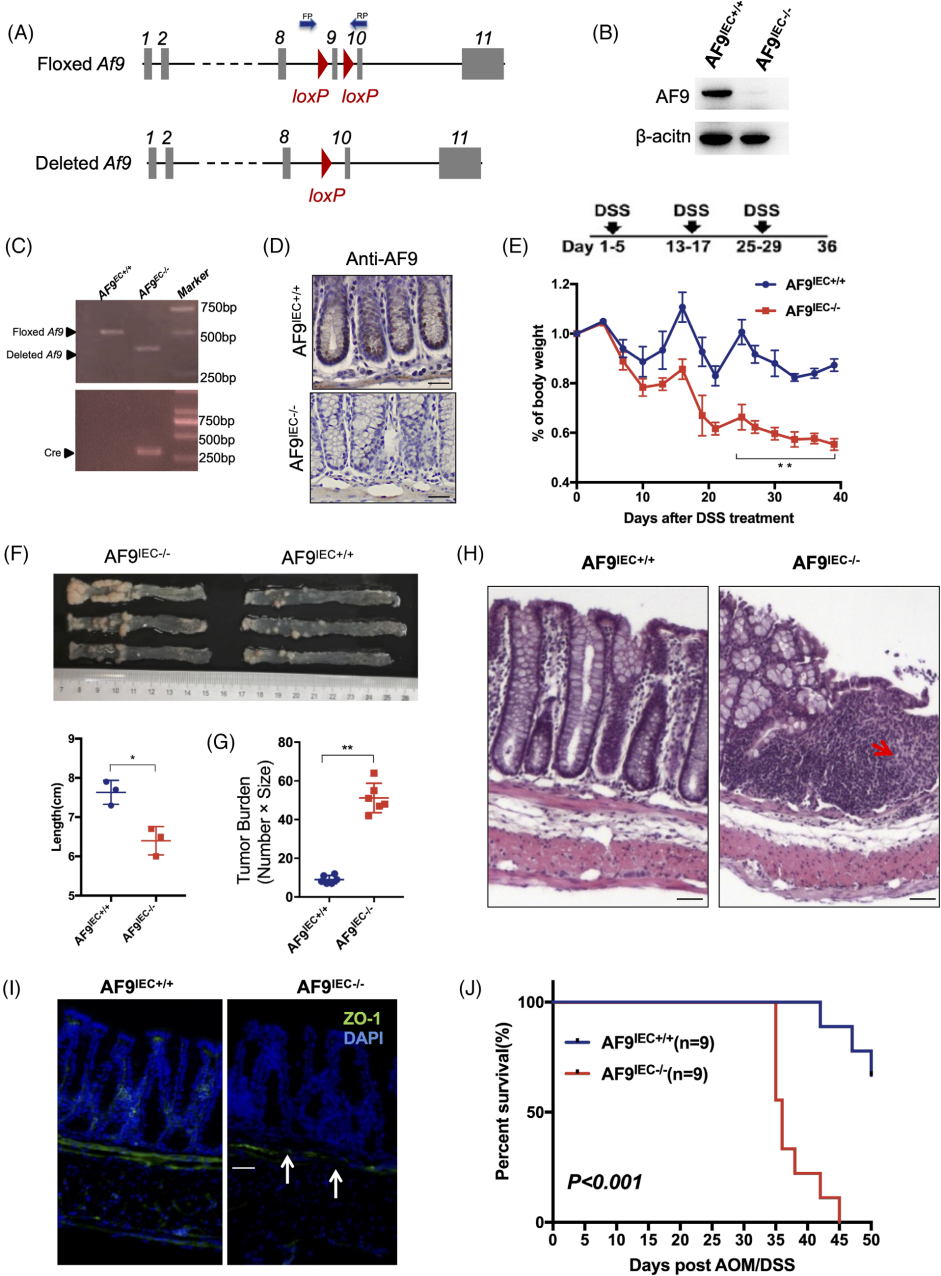
AF9 suppresses cancer initiation and progression in AOM/DSS induced CRC model**. (**A) Scheme of the *Af9* gene locus and related alleles. The *Af9*‐floxed alleles have two loxP sites (red triangles) flanking the ninth exon (grey boxes). Mice with *Af9*‐floxed alleles were crossed with a *Cre* line to generate the deleted *Af9* allele. (B) Western blot analysis of colon tissues from *AF9^IEC+/+^* and *AF9^IEC‐/−^* mice using indicated antibodies. (C) Genotypes were determined by PCR using genomic DNA from intestinal epithelial cells. (D) *AF9^IEC‐^* ^/−^ was identified by IHC using antibody against AF9. (E) Body weight of *AF9^IEC+/+^* and *AF9^IEC‐/−^* mice was measured after treatment with DSS. Body weight changes in the mice during three cycles of 3% DSS treatment. (F and G) The mice were euthanised to measure colon length (*n* = 6) and tumour burden (*n* = 6) on Day 36. (H) Hematoxylin and eosin (H&E)‐stained sections of middle–distal colon tissue. Arrows, infiltration of immune cell and epithelial cell damage. (I) Representative images of ZO‐1 staining in colon sections of mice treated with DSS. (J) Kaplan–Meier plot showing the survival of AF9^IEC+/+^ and AF9^IEC‐/−^ mice after AMO/DSS treatment (*p < .05, **p < .01).

To identify the suppressive role of AF9 in CRC, we further applied AOM/DSS‐induced CRC model to test whether depletion of AF9 in intestine epithelial cells could block or delay CRC initiation or progression. The method of AOM/DSS‐induced mouse model was previously described in our own work.^28^ The strategy of knockout (KO) *AF9* in intestine epithelial cells is shown in Figure 3A. The successful KO mice of AF9 in intestine epithelial cells (called *AF9^IEC‐/−^*) was confirmed by PCR, western blot and IHC (Figure 3C,D). We recorded the body weight of mice after DSS treatment and observed that *AF9^IEC‐/‐^* mice lost weight faster, suggesting AF9 could confer tolerance to DSS (Figure 3E). We sacrificed AOM/DSS‐treated mice and stripped the colorectum and found that *AF9^IEC‐/‐^* mice had a shorter colorectal length, which may be caused by stronger inflammation (Figure 3F). Also, *AF9^IEC‐/‐^* mice had much more tumours in the intestines (Figure 3G).

As the CRC progression would form solid tumours and destroy the basement membrane, we use H&E staining to identify the solid tumour and ZO‐1^29^, ^30^ staining to prove the destruction of the basement membrane. Data showed loss of AF9 would accelerate the tumour formation (Figure 3H,I). The survival duration of mice with or without AF9 in IEC presented a big difference: *AF9^IEC‐/‐^* mice had a short life after AOM/DSS/ (Figure 3J). Thus, we preliminarily conclude AF9 suppresses cancer initiation and progression in AOM/DSS‐induced CRC model.

### 3.4 miR‐145 targets AF9 3′UTR and silences AF9 mRNA

**FIGURE 4:**
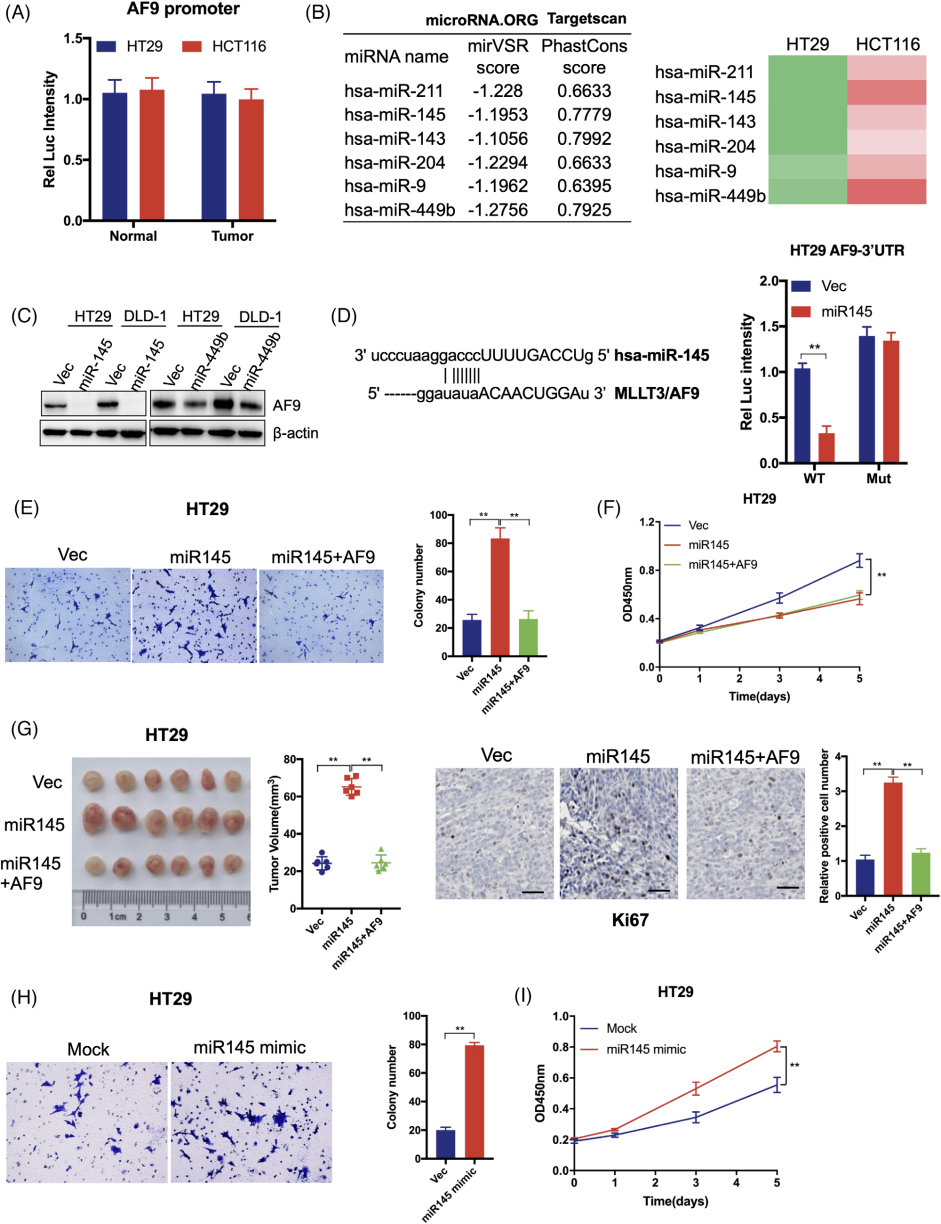
miR‐145 targets AF9 3′ untranslated region (3′UTR) and silences AF9 mRNA. (A) Dual‐luciferase reporter assay was performed to test the AF9 promoter activity. (B) microRNA.ORG and Targetscan were used to predict the potential miRNA, which could target 3′UTR of homo sapiens AF9. The green represents low expression of miRNAs, while the red represents high expression of miRNAs. And the deeper the colour is, the higher or lower expression of miRNAs is. (C) miR145 or miR449b was transfected into HT29 or DLD‐1 cells. Western blot analysis was used to detect AF9 protein level by indicated antibodies. (D) Dual‐luciferase reporter assay was performed to test the activity of wild type or mutant 3′UTR by co‐transfection with miR‐145. E, Representative images of transwell assays performed in HT29 cells (including Vec, miR‐145 or miR‐145+AF9) are shown (scale bar represents 60 μm). (F) Cell proliferation of HT29 cells (including Vec, miR‐145 or miR‐145+AF9) was measured by CCK‐8. (G) Xenograft formation. HT29 cells (including Vec, miR‐145 or miR‐145+AF9) were implanted into left groin of nude mice (*n* = 6 for each group). The tumour volume was measured at the end of the experiment. Ki67 staining was performed to test the in vivo proliferation. (H) Representative images of transwell assays performed in HT29 cells transfected with or without miR‐145 mimic are shown (scale bar represents 60 μm). (I) Cell proliferation of HT29 cells transfected with or without miR‐145 mimic was measured by CCK‐8 (**p < .01).

We examined the promoter activity of AF9 gene by introducing its promoter from a tumour or normal tissue into pGL3B and found that the promoter activity of the AF9 gene in tumour or normal tissue has no difference (Figure 4A). It is predicted that miRNA accounts for 1%−5% of the human genome and regulate sat least 30% of mRNA mature.^31^ To understand how AF9 is silenced in tumours, we predicted the potential miRNAs that could target AF9 mRNA as shown in Figure 4B; six miRNAs may be involved in regulating AF9 mRNA. Overall, these 6 miRNAs expressed high in HCT116, but low in HT29 (Figure 4B). We selected two miRNAs: miR‐145 and miR‐449b for further research due to their greater difference in HT29 and HCT116 cells. When we forced the expression of miR‐145 or miR‐449b in HT29 or DLD‐1 cells, only miR‐145 dramatically reduced AF9 expression (Figure 4C). We showed the predicted region of AF9 3′UTR targeted by miR‐145 and then mutated this region from AGGUCAA to AGCGCAA. After mutating, miR‐145 could not affect AF9 3′UTR any more, proving that miR‐145 regulates AF9 mRNA stability by targeting its 3′UTR (Figure 4D). In order to investigate the effect of miR‐145 on CRC cells, we overexpressed miR‐145 in HT29 and DLD‐1 cells, and based on this, forced expression of AF9 through the Cytomegalovirus (CMV) promoter (Supporting Information Figure S2A). As shown in Figure 4E,F, miR‐145 overexpression in HT29 increased cell migration and proliferation, which could be impaired by exogenous expression of AF9 (Figures 4E,F and S2B,C). Consistently, using the xenograft model, the enhanced capacity of cell proliferation acquired from miR‐145 overexpression could be hampered by the forced expression of AF9 (Figure 4G). Further, miR‐145 mimic was transiently introduced into CRC cells to inhibit AF9 mRNA expression. A consistent result with Figure 4E,F was observed that miR‐145 mimics impaired AF9 expression (Supporting Information Figure S2D) and promoted cell proliferation and migration (Figures 4H,I and S2E,F). Hence, AF9 mRNA was silenced by miR‐145 in CRC cells.

### 3.5 AF9 positively regulates the expression of PCK2 and FBP1

**FIGURE 5:**
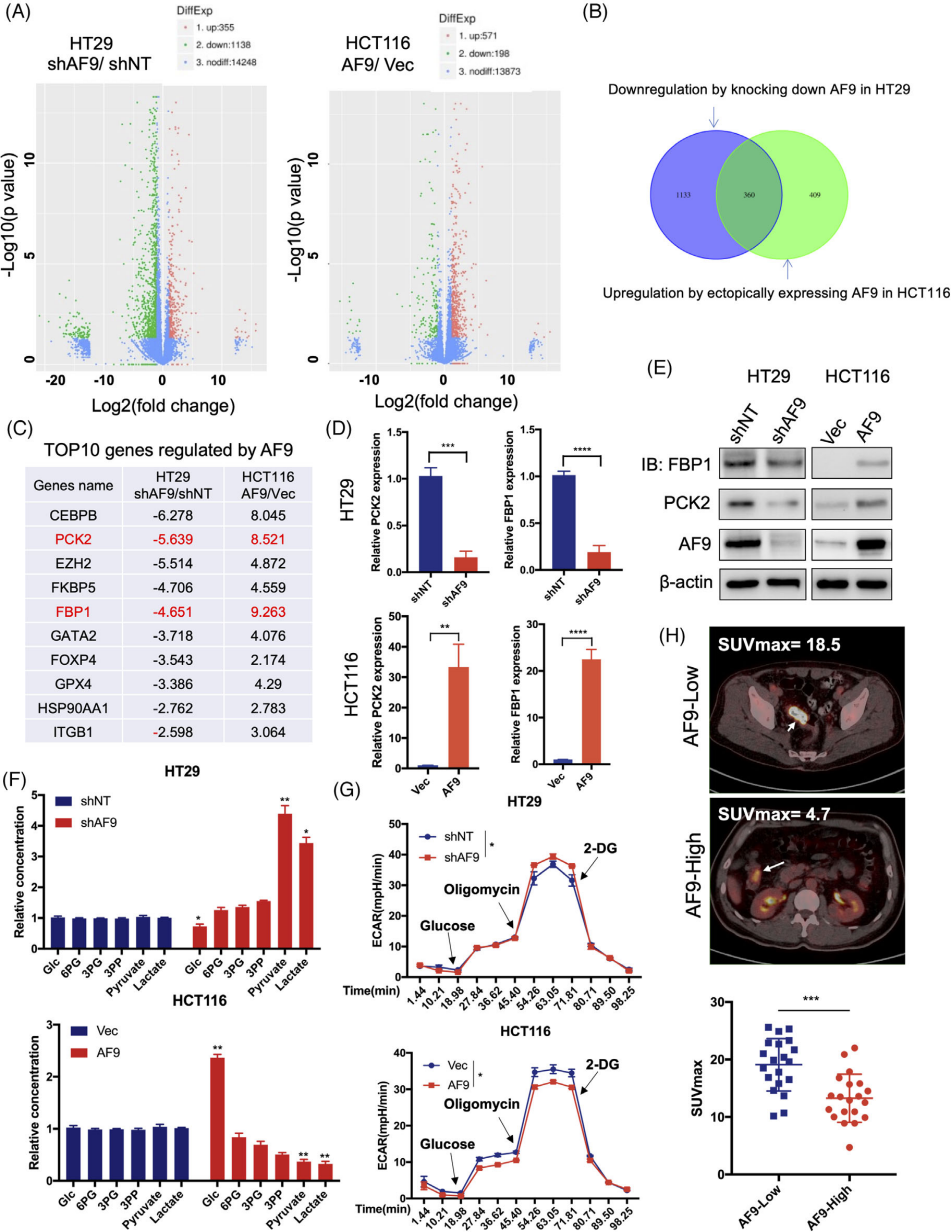
AF9 positively regulates the expressions of phosphoenolpyruvate carboxykinase 2 (PCK2) and FBP1, then hampers glycolysis. (A) RNA‐seq analysis of HT29 cells with or without depleting AF9 or HCT116 cells with or without forced expression of AF9. (B) AF9 positively regulated genes were found in HT29 and HCT116 cells. (C) The name and ratio value of TOP10 genes positively regulated by AF9 were listed. (D and E) quantitative real‐time PCR and western blot verified the expression of PCK2 and FBP1 in HT29 cells with or without depleting AF9 or HCT116 cells with or without forced expression of AF9. (F) The key metabolites in the glycolytic pathway were quantified by mass in HT29 cells with or without depleting AF9 or HCT116 cells with or without forced expression of AF9. (G) The cells were cultured with the cell density reaching 50%, and extracellular acidification rate (ECAR) was measured by the XF glycolysis stress test. (H) Representative fluoro2‐D‐deoxyglucose F18 (18F‐FDG) PET/computed tomography images of CRC patients with low or high AF9 expression; difference analysis of standardised uptake value (SUV)max in the AF9‐high and AF9‐low groups (*p < .05, **p < .01, ***p < .001, ****p < .0001).

To understand how AF9 could inhibit tumourigenesis, we use RNA‐seq to profile the genes regulated by AF9. As shown in Figure 5A, the knockdown of AF9 in HT29 leads to the downregulation of 1138 genes and upregulation of 355. Forced expression of AF9 in HCT116 leads to an upregulation of 571 and a downregulation of 198 (Figure 5A). We analysed AF9‐regulated genes in HT29 and HCT116 cells and found that 360 genes were regulated by AF9 in both cells (Figure 5B). KEGG results indicated that these genes were mainly enriched in Hypoxia‐induced factor 1 (HIF‐1) signalling pathway, glycolysis/gluconeogenesis, taurine and hypotaurine metabolism (Supporting Information Figure S3A). Among these genes, we found PCK2 and FBP1, the two glucogenic genes, were listed in the TOP10 genes regulated by AF9 (Figure 5C). Next, we tested the expression of PCK2 and FBP1 in the cells expressing shNT and shAF9 or Vec and AF9. Generally, the loss of AF9 reduced the expression of PCK2 and FBP1, while forced expression of AF9 could reinforce PCK2 and FBP1 (Figures 5D and S3B). The consequent protein level showed the consistent correlation between AF9 and PCK2, FBP1 (Figures 5E and S3C). As AF9 could upregulate or maintain glucogenesis, we determined the inner concentration of Glc, 6PG, 3PG, 3PP, pyruvate and lactate in the above cells. Overall, loss of AF9 reduced the concentration of Glc and 6PG but increased the concentration of the other four metabolites, while forced expression enhanced the concentration of Glc and 6PG but decreased the concentration of the other four metabolites, indicating tumour cells have the suppressed glycolysis after restored AF9 expression (Figure 5F and S3D). Through ECAR, we observed that the level of glycolysis in HT29 and DLD‐1 cells increased accompanying the depletion of AF9, while the level of glycolysis decreased when AF9 was reinforced in HCT116 and RKO cells (Figures 5G and S3E). These indicate that in CRC cells, PCK2 and FBP1 could be regulated by AF9 to promote the glycolysis metabolism in CRC. Furthermore, we analysed the 18F‐FDG PET/CT results in 40 CRLM patients, and we found that the SUVmax values in the AF9‐low group (19.1 ± 1.021, *n* = 20) were significantly higher than those in the AF9‐high group (13.27 ± .9347, *n* = 20), indicating enhanced glucose metabolism in tumour tissues with AF9 downregulation (Figure 5H).

### 3.6 AF9 targets PCK2 and FBP1 by recognising H3K9ac but not H3K18ac

**FIGURE 6:**
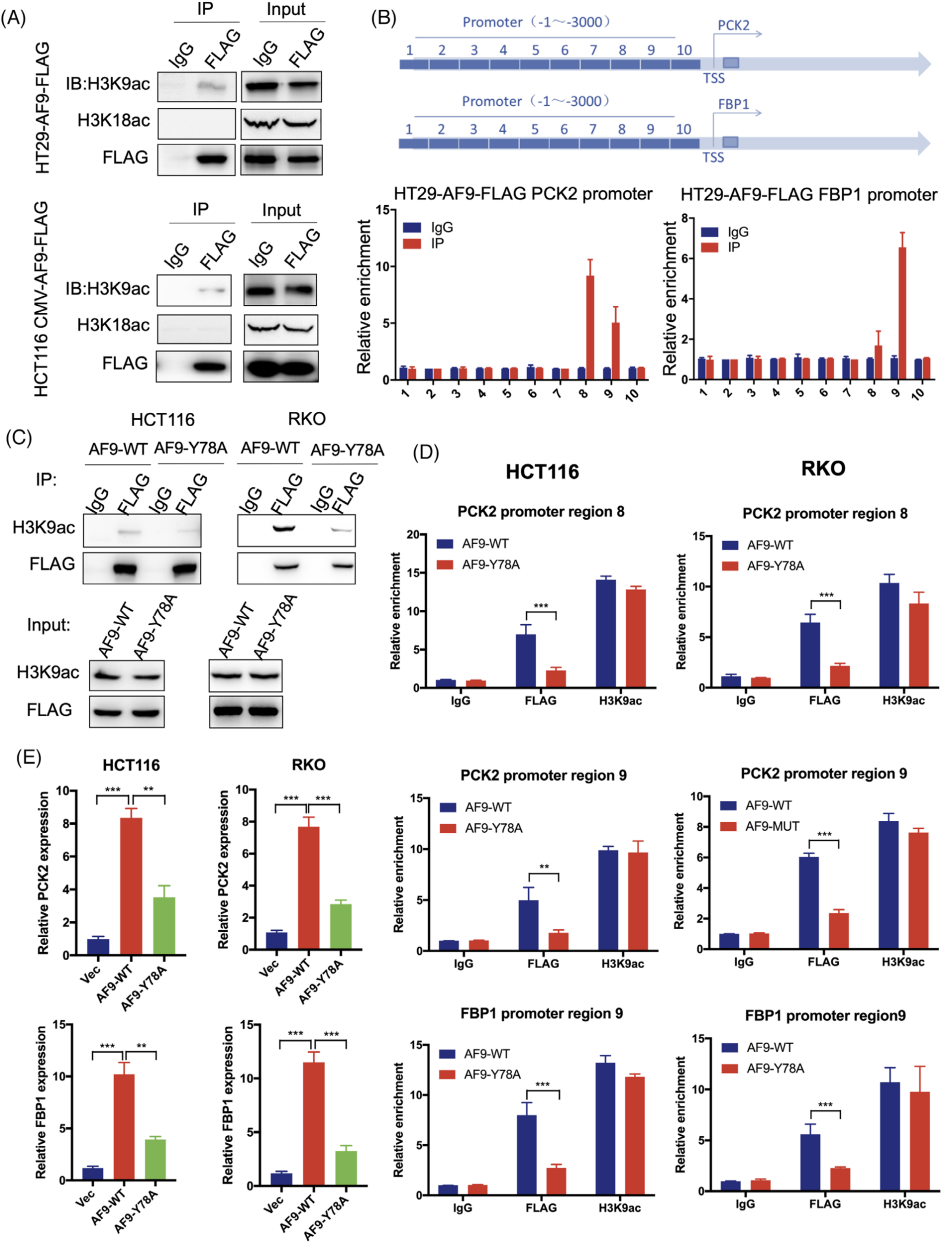
AF9 targets PCK2 and FBP1 through H3K9 acetylation (H3K9ac) but not H3K18ac. (A) Co‐immunoprecipitation (IP) was performed to test the association between AF9 and H3K9ac, H3K18ac in HT29 and HCT116 cells. (B) Chip assay was performed to test AF9 targeting the regions of PCK2 and FBP1 promoters. (C) Co‐IP was performed to test the association between AF9 and H3K9ac in HCT116 and RKO cells expressing wild type or mutant AF9. AF9 mutant: Y78A mutation. (D) In HCT116 and RKO cells, ChIP assay was performed to test wild type or mutant AF9 targeting the promoter of PCK2 and FBP1. (E) PCK2 and FBP1 expressed in HCT116 and RKO cells expressing wild type or mutant AF9. Vec was used as an empty control (**p < .01, ***p < .001).

Histone mark reader proteins function as adaptors for gene expression.^32^, ^33^ Histone acetylation at lysine residues was well‐established to promote gene expression. AF9 recognises H3K9ac and H3K18ac meanwhile.^34^ To understand the association of AF9 with H3K9ac and H3K18ac in CRC, we first knocked a FLAG into the C terminal of AF9 to construct a cell line: HT29‐AF9‐FLAG. We used antibody against FLAG to perform ChIP. We performed a co‐immuno‐precipitation assay. As shown in Figure 6A, AF9 tights with H3K9ac, but not H3K18ac, indicating in CRC, AF9 could activate gene expression by recognising H3K9ac (Figure 6A). To confirm the specificity of antibody applied in ChIP assay, we depleted AF9 in HT29‐AF9‐FLAG cells (Supporting Information Figure S4A). qPCR identified AF9 could bind the promoter of PCK2 and FBP1 meanwhile, which could explain that AF9 maintains the expression of PCK2 and FBP1(Figure 6B). As shown in Supporting Information Figure S6B, the loss of AF9 heavily reduced the AF9 targeting the promoter of PCK2 and FBP1 (Supporting Information Figure S4B). AF9 YEATS domain recognises H3K9ac using a hydro‐bond net and mutating Y78 to A78 could abolish the binding of YEATS to H3K9ac.^35^ In this study, Y78A mutant lost the association with H3K9ac (Figure 6C,D). As a result, Y78A mutant impaired the expression of PCK2 and FBP1 in CRC cells (Figure 6E).

### 3.7 PCK2 and FBP1 restore the suppressive capacity of AF9

**FIGURE 7:**
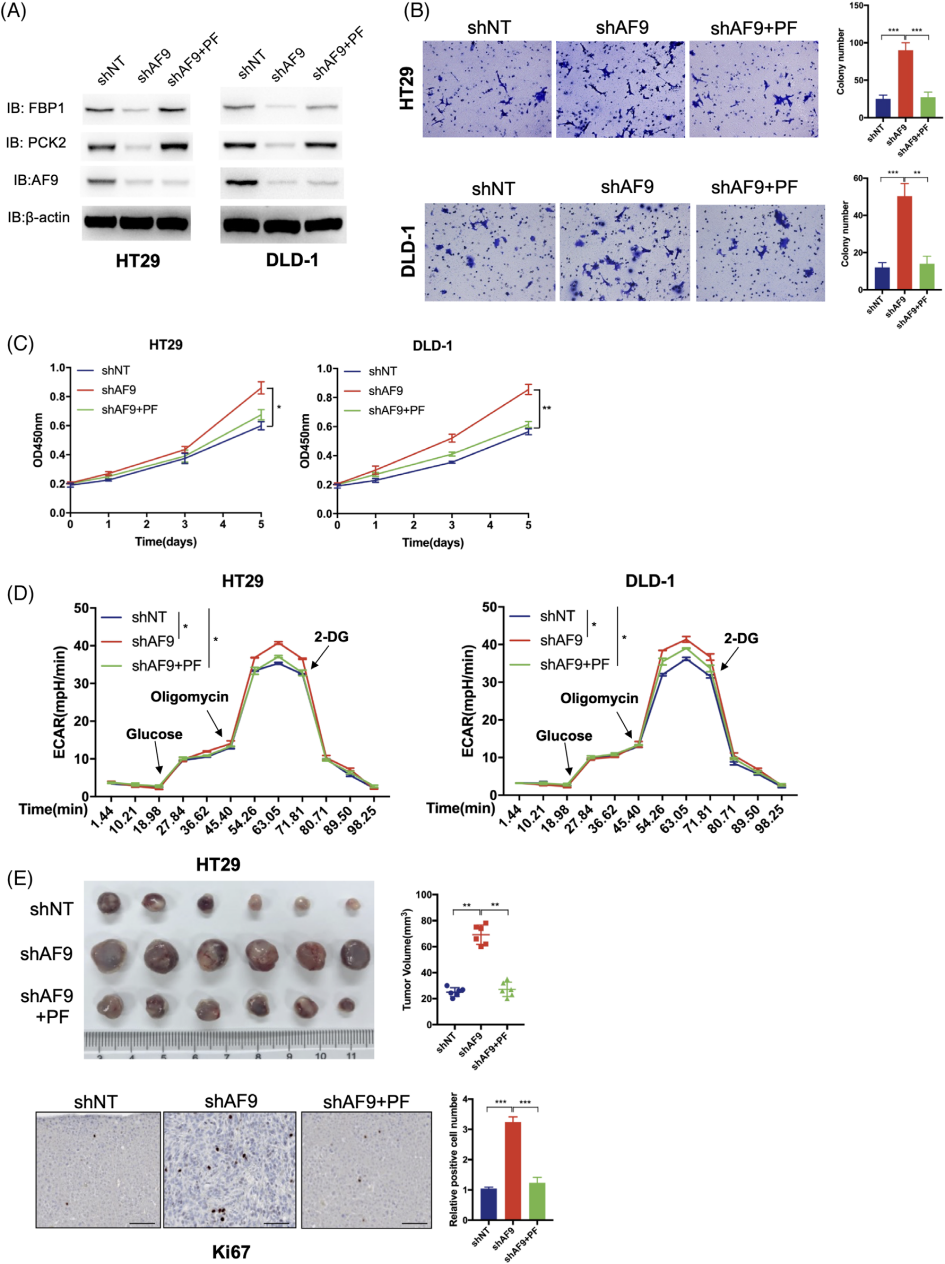
Forced expression of PCK2 and FBP1 restored the suppressive capacity of AF9. (A) Successful restored expression of PCK2 and FBP1 meanwhile in HT29 and RKO cell with depletion of AF9. PF: PCK2+FBP1. (B) Representative images of transwell assays performed in HT29 and DLD‐1 cells (including shNT, shAF9 and shAF9+PF) are shown (scale bar represents 60 μm). (C) Cell proliferation of HT29 and DLD‐1 cells (including shNT, shAF9 and shAF9+PF) was measured by CCK‐8. (D) The HT29 and DLD‐1 cells (including shNT, shAF9 and shAF9+PF) were cultured with the cell density reaching 50%, and ECAR was measured by the XF glycolysis stress test. (E) Xenograft formation. HT29 cells (including shNT, shAF9 and shAF9+PF) were implanted into left groin of nude mice (*n* = 6 for each group). The tumour weight was measured at the end of the experiment. Ki67 staining was performed to test the in vivo proliferation (*p < .05, **p < .01, ***p < .001).

**FIGURE 8:**
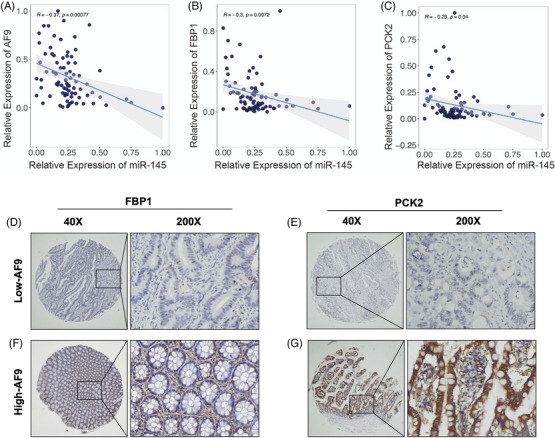
The AF9 expression was significantly associated with miR145, FBP1 and PCK2. (A, B and C) Pearson correlation analysis showing the correlation between miR‐145 and AF9, miR‐145 and FBP1, miR‐145 and PCK2. (D and E) Representative IHC images of FBP1 and PCK2 with high AF9 expression. (F and G) Representative IHC images of FBP1 and PCK2 with low AF9 expression.

To confirm PCK2 and FBP1 work as the downstream genes of AF9, we resumed PCK2 and FBP1 expression in HT29 and DLD‐1 cells with the knocking down of AF9 (Figure 7A). As expected, when we forced expression of PCK2 and FBP1 meanwhile, CRC cells restrained the proliferation and migration that was released by loss of AF9 (Figure 7B,C). To test whether forced expression of PCK2 and FBP1 in cells with AF9 depletion was associated with alterations in cellular metabolism, we measured their ECAR and found the burst glycolysis by depletion of AF9 was partially inhibited by PCK2 and FBP1 (Figure 7D). Xenograft implantation showed the accelerated formation of solid tumours after AF9 depletion could be dampened by restored expression of PCK2 and FBP1 meanwhile (Figure 7E, left panel). The proliferation rate of tumour cells was measured by Ki67, and a consistent result with in vitro cell proliferation assay in Figure 7B was observed (Figure 7E, right panel). Clinically, we found that AF9 expression was negatively associated with the expression of miR145 but positively with FBP1 and PCK2. High expression of AF9 was correlated to low level of miR145 and high level of FBP1 and PCK2, and vice versa (Figure 8).

## 4 DISCUSSION

Epigenetic deregulation, especially mutagenesis or the abnormal expression of ERFs, is known to play a significant role in the development and progression of malignant tumours.^36^ ERFs are a diverse group of molecules that control the activity of genes by modifying the structure of chromatin, the complex of DNA and proteins in the nucleus. By modifying the chromatin structure, ERFs can either promote or suppress the expression of specific genes, thereby influencing critical cellular processes involved in cancer progression, such as cell growth, differentiation and metastasis. Aberrant alterations in ERFs can disrupt the normal epigenetic landscape and lead to widespread changes in gene expression patterns, contributing to the initiation and maintenance of cancer cells. Understanding the role of ERFs in cancer progression is crucial for uncovering potential therapeutic targets and developing strategies to restore normal epigenetic regulation in cancer cells.^37^, ^38^ Through our siRNA library screening in 591 ERFs coding genes, we found that the deletion of AF9 could promote the progression of CRC cells. In our study, we revealed that AF9 served as a tumour suppressor in CRC, and its downregulation was negatively associated with the prognosis of CRC patients.

Usually, cancer‐associated genes are divided into oncogenes or tumour suppressor genes depending on their pro‐ or anti‐function in the progression of the tumour.^39^ However, it is not a novelty that many genes have a dual role in the progression of different cancers and so as AF9. The most well‐known pro‐tumour effect of AF9 is its involvement in chromosomal translocations with the MLL gene, leading to fusion oncogenes. These fusion proteins, such as MLL‐AF9, have potent oncogenic properties and contribute to the development of leukaemia. They disrupt normal gene regulation, promote uncontrolled cell proliferation, impair differentiation and drive leukaemic transformation.^40^ Also, in liver cancer, AF9 was proved to be an oncogene whose expression increased as the tumour stage progressed.^23^ However, a previous study has also revealed that AF9 acted as a tumour suppressor in breast cancer to negatively regulate the proliferation of breast cancer by inhibiting cell cycle progression, and its signalling pathway miR‐5694/AF9/Snail could be applied as a therapeutic target.^41^ Therefore, it is not unusual to find that AF9 functioned as a tumour suppressor in CRC.

The increased rate of glycolysis is a common metabolic alteration observed in cancer. In CRC, it has been noted that active glycolysis is achieved through the upregulation of glycolytic enzymes and transporters.^42^ Certain glycolytic enzymes and transporters have been shown to contribute to the proliferation and metastasis of CRC.^43^ In general, most steps of gluconeogenesis can be considered the reverse process of glycolysis, involving multiple steps from glucose phosphorylation to pyruvate generation.^44^ In normal tissues, cells can utilise pyruvate as a precursor and undergo the exact opposite process of glycolysis to produce glucose.^45^, ^46^, ^47^ Thus, gluconeogenesis is regarded as a natural metabolic pathway capable of counteracting the rapid progression and deterioration of tumours.^44^ Unfortunately, in tumour cells, the expression levels of enzymes involved in the gluconeogenesis pathway, such as FBP1/2 and PCK1/2, are low or even silenced. Targeting key enzymes involved in gluconeogenesis holds promise as a therapeutic strategy to impair CRC cell survival and inhibit tumour growth.^5^ Discovering the mechanisms responsible for the silencing of metabolic enzymes in the gluconeogenesis pathway is a crucial prerequisite for restoring gluconeogenesis to combat glycolysis in CRC. In our study, the low expression of AF9 could inhibit the expression of gluconeogenic genes, FBP1 and PCK2.

Recent studies have revealed intriguing insights into the molecular mechanisms underlying the function of AF9 in CRC. AF9 has been shown to exhibit a selective recognition of histone modifications in CRC cells. Specifically, AF9 demonstrates a strong affinity for H3K9ac, while its association with H3K18ac is absent.^42^ This finding suggests that the YEATS domain of AF9 plays a critical role in determining its target specificity by forming complexes with other proteins within the cellular environment. Furthermore, it has been observed that AF9's interaction with H3K9ac is closely linked to the regulation of gene expression in CRC. Notably, AF9 relies on its recognition of H3K9ac in the promoter region to facilitate the upregulation of key genes such as PCK2 and FBP1. Through this mechanism, AF9 exerts its ability to activate gene expression and contribute to cellular processes associated with CRC progression. Interestingly, the regulatory function of AF9 extends beyond its YEATS domain. It has been proposed that AF9 requires the involvement of chaperone proteins to exert specific regulation and orchestrate its gene regulatory activities effectively. These chaperone proteins likely facilitate the assembly of protein complexes, enabling AF9 to achieve precise and context‐dependent gene regulation.

The intricate interplay between AF9, histone modifications and chaperone proteins in CRC cells highlights the complexity of AF9‐mediated transcriptional regulation. Further investigations are warranted to elucidate the precise mechanisms by which AF9 forms complexes, recruits chaperones and orchestrates gene expression programs in CRC. Understanding these mechanisms will not only deepen our knowledge of AF9's role in CRC pathogenesis but may also pave the way for potential therapeutic interventions targeting AF9‐associated dysregulation in CRC.

## 5 CONCLUSION

The current study revealed a novel tumour suppressor, AF9, which was significantly downregulated in CRC tissue and was negatively associated with the prognosis of CRC patients. Downregulation of AF9 inhibited the expression of gluconeogenic genes, FBP1 and PCK2, through recognising H3K9ac, thus suppressing CRC proliferation, migration and glucogenesis. Meanwhile, miR‐145 targeted AF9 mRNA to downregulate its expression. These observations indicated that disrupting the miR‐145/AF9 axis could be a potential strategy to maintain glucogenesis in normal tissues.

## CONFLICT OF INTEREST STATEMENT

The authors declare no potential conflicts of interest.

## FUNDING INFORMATION

The National Natural Science Foundation of China (81972185, 81972293); ‘Dawn’ Program of Shanghai Education Commission, China (21SG09); Shanghai Rising‐Star Program (19QA1402200); Shanghai Science and Technology Innovation Action Plan ‘Outstanding Academic Leader Program’ (22XD1420500).

## Supporting information

Supporting Information

Supporting Information

Supporting Information

Supporting Information

Supporting Information

Supporting Information

## Data Availability

The datasets used and/or analysed during the current study are available from the corresponding author on reasonable request.
