# Supplementary figures and images for: AF9 sustains glycolysis in colorectal cancer via H3K9ac‐mediated PCK2 and FBP1 transcription
Source: Clin Transl Med. 2023 Aug 10;13(8):e1352. doi: 10.1002/ctm2.1352 (PMC10413954; doi:10.1002/ctm2.1352)

Sup Figure 1

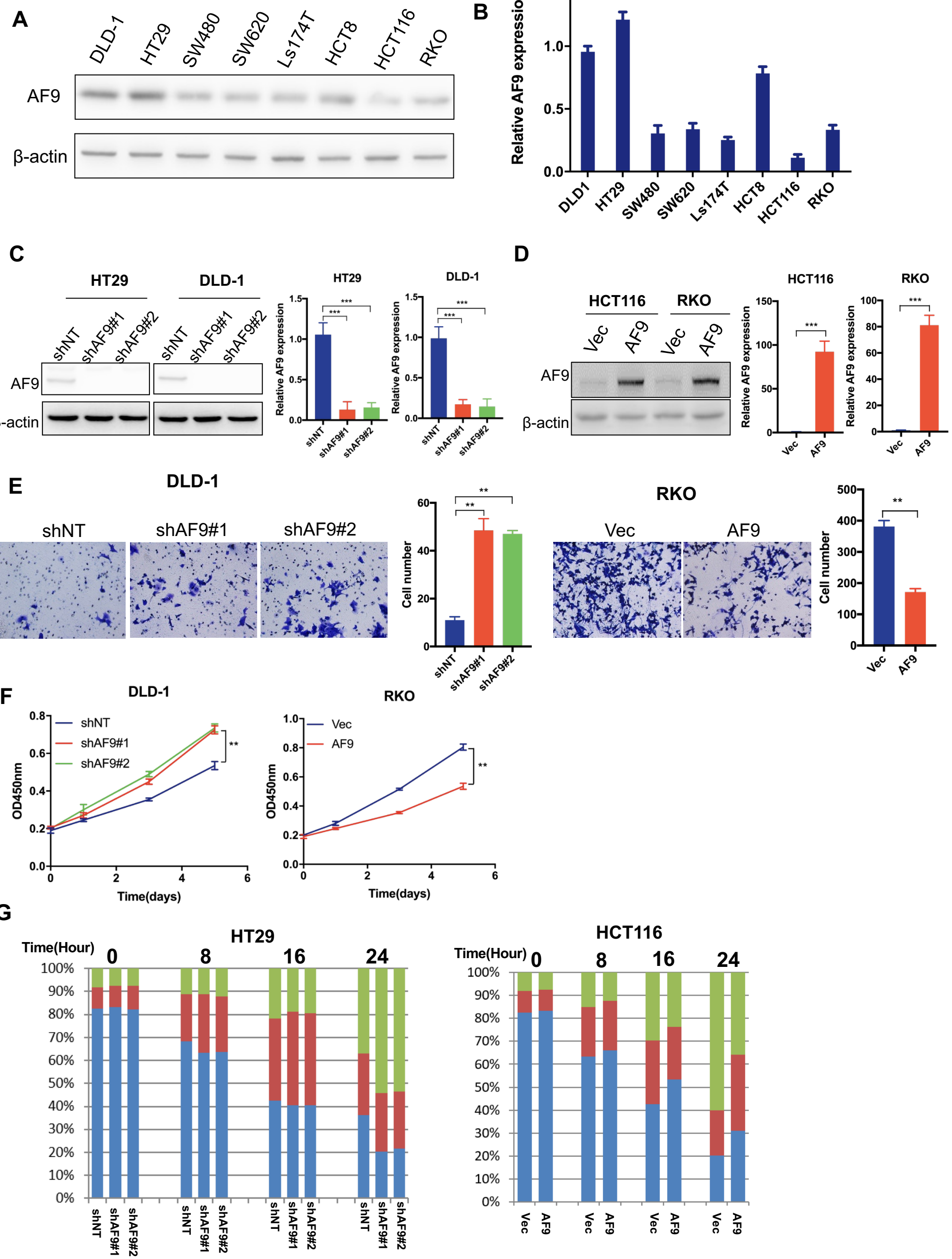

Supplement: Supplementary file 1 — Supporting Information [file CTM2-13-e1352-s005.pdf]

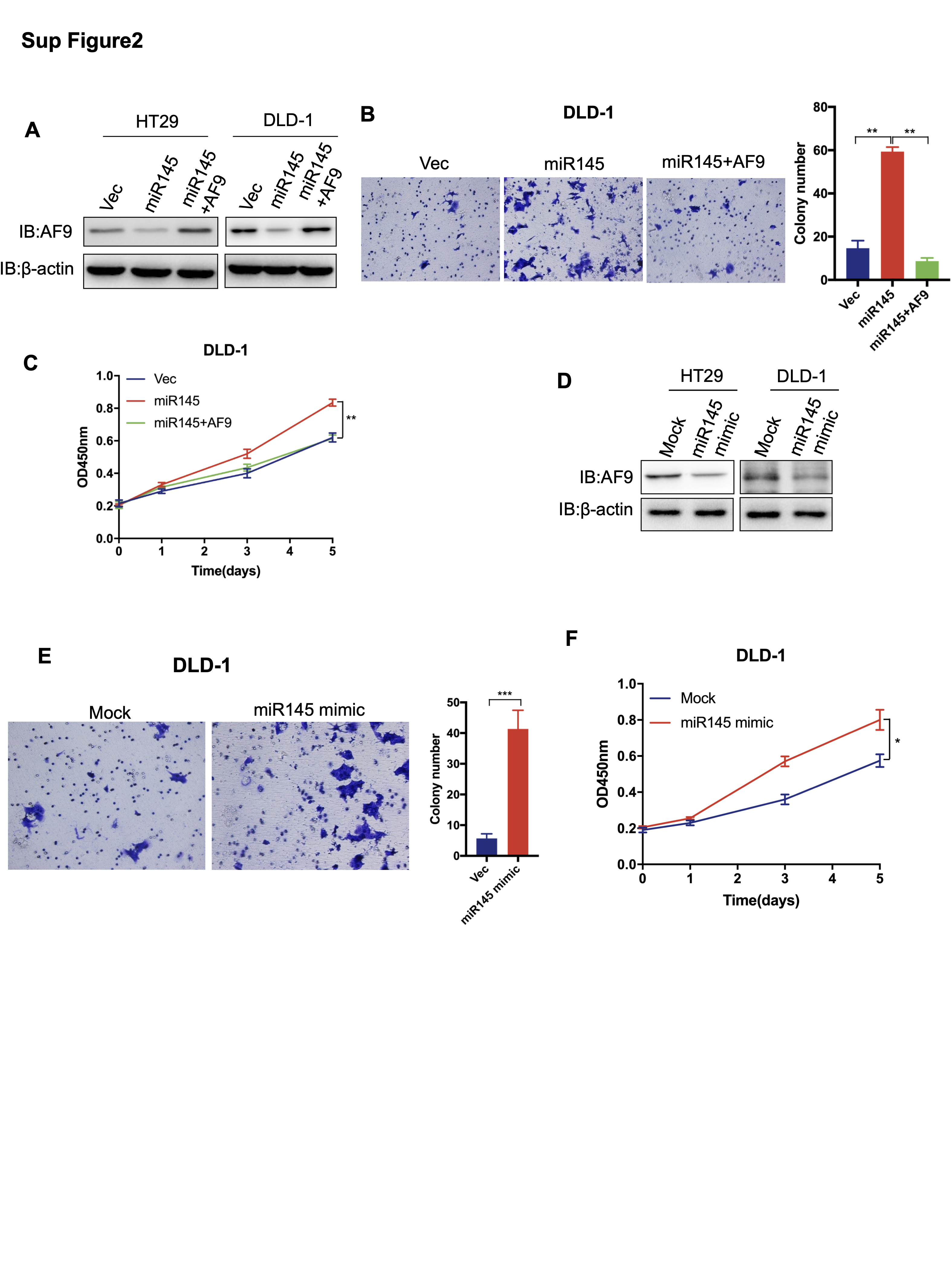

Supplement: Supplementary file 2 — Supporting Information [file CTM2-13-e1352-s006.tiff]

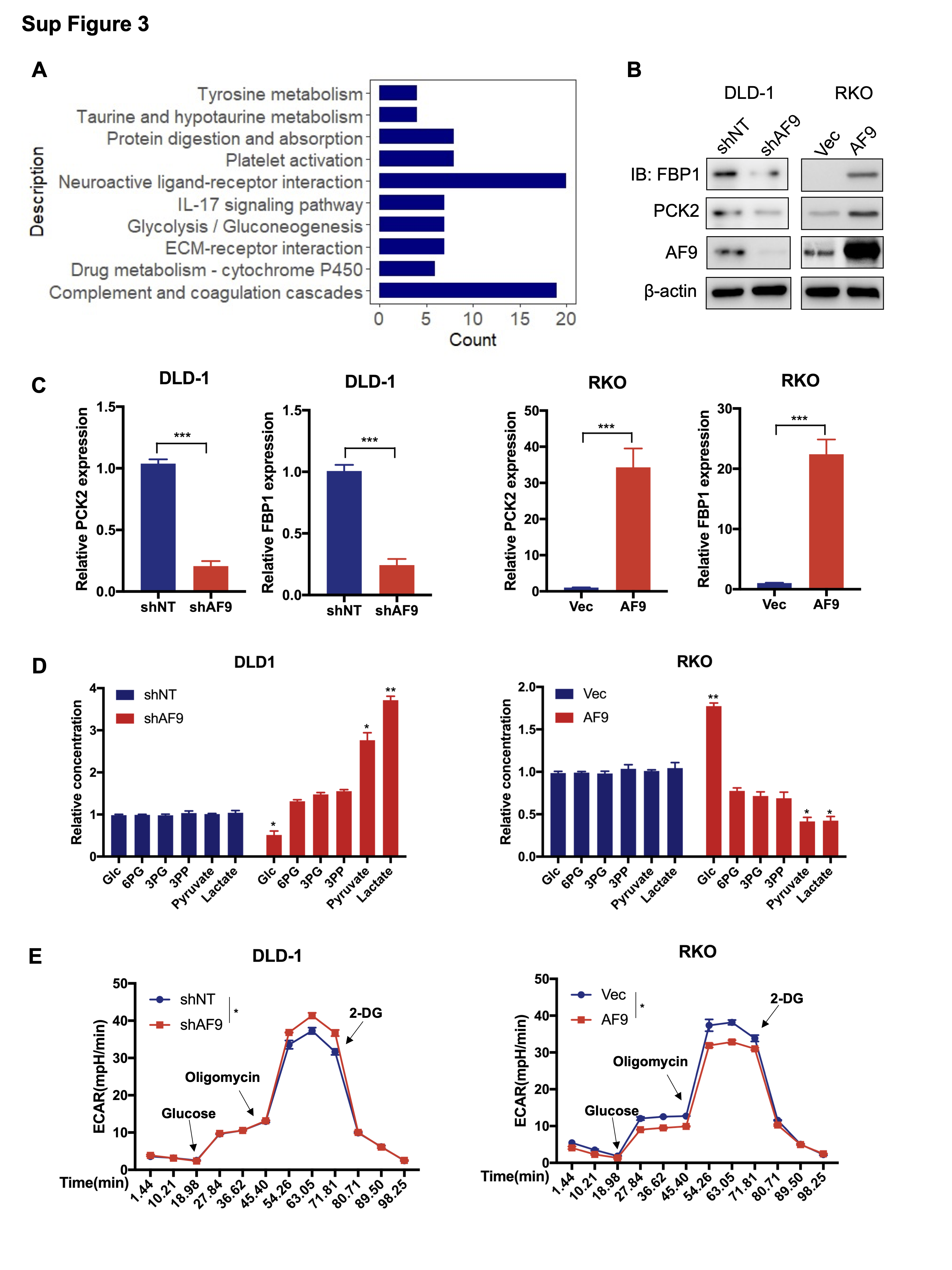

Supplement: Supplementary file 3 — Supporting Information [file CTM2-13-e1352-s001.tiff]

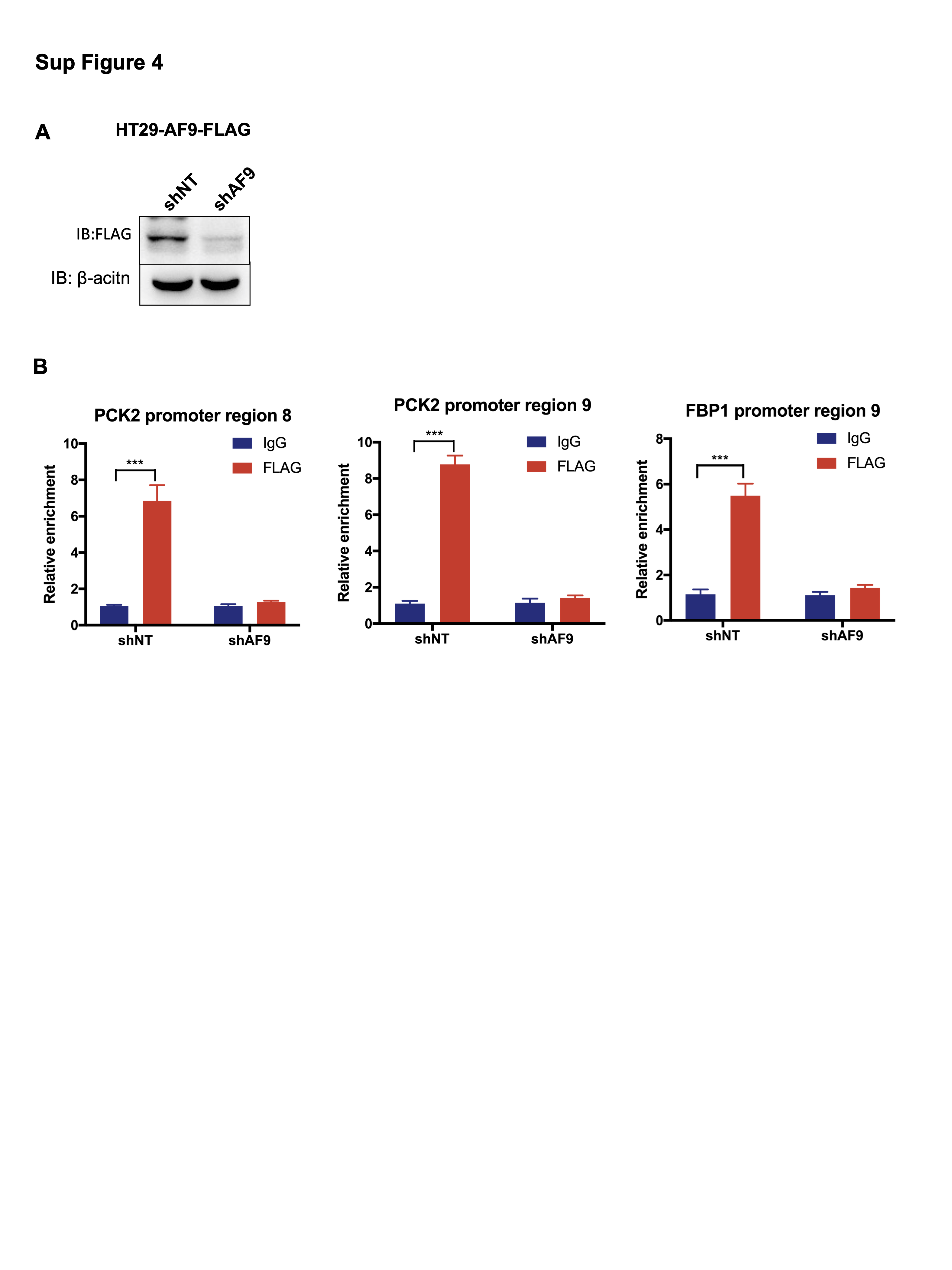

Supplement: Supplementary file 4 — Supporting Information [file CTM2-13-e1352-s004.tiff]

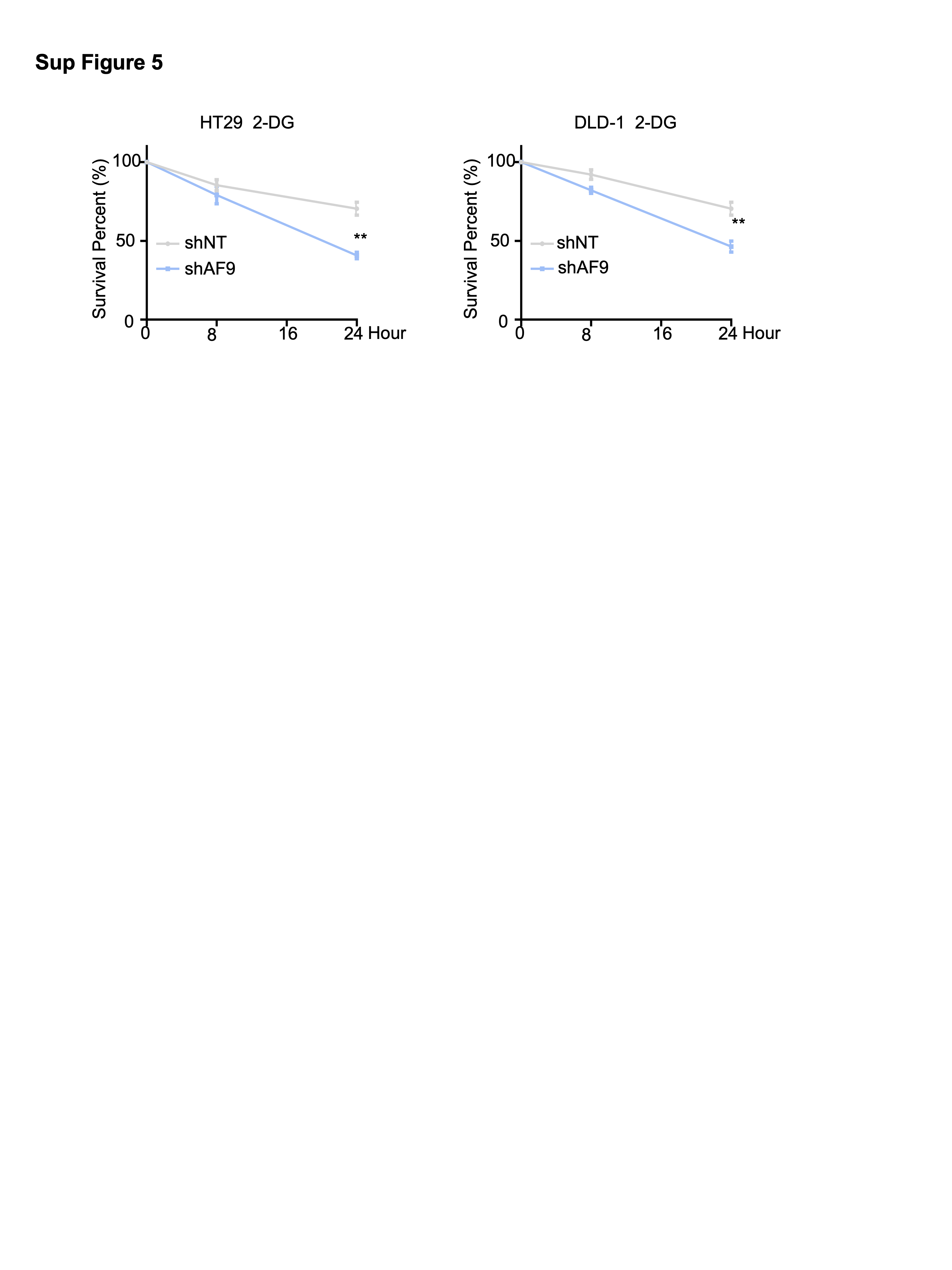

Supplement: Supplementary file 5 — Supporting Information [file CTM2-13-e1352-s003.tiff]
