# Supplementary material for: AF9 sustains glycolysis in colorectal cancer via H3K9ac‐mediated PCK2 and FBP1 transcription
Source: Clin Transl Med. 2023 Aug 10;13(8):e1352. doi: 10.1002/ctm2.1352 (PMC10413954; doi:10.1002/ctm2.1352)
Supplement: Supplementary file 6 — Supporting Information [file CTM2-13-e1352-s002.docx]

**Supplementary Materials**

| **Supplementary Table** | |
| --- | --- |
| PCR primers used for constructs | |
| pCDH-AF9 ORF-F | ATGGAATTCGCTAGCGGATCC atggctagctcgtgtgccgtg |
| pCDH-AF9 ORF-R | ATGTCGACCTCGAGTGCGGCCGCggatgttccagatgtttccag |
| pGL3B-AF9 promoter-F | ATGGAATTCGCTAGCGGATCCtgtaaaggaagccagtagttgata |
| pGL3B-AF9 promoter-R | ATGTCGACCTCGAGTGCGGCCGCtccagggtaaagaagat gatt |
| pCDH-miR-145-F | ATGGAATTCGCTAGCGGATCCaaggccactcgctcccaccttg |
| pCDH-miR-145-R | ATGTCGACCTCGAGTGCGGCCGCggcaaatccagctgtgaaac |
| pCDH-miR-449b-F | ATGGAATTCGCTAGCGGATCC tgtgtgatgagtggcagtgt |
| pCDH-miR-449b-R | ATGTCGACCTCGAGTGCGGCCGcaccagctaacatacactgcca |
| qPCR primers for testing gene expression | |
| AF9-F | 5’- TTTGTGGAGAAAGTCGTCTTCC |
| AF9-R | 5’- GAGGTGATTCACTGGTGGATG |
| FBP1-F | 5’- CGCGCACCTCTATGGCATT |
| FBP1-R | 5’- TTCTTCTGACACGAGAACACAC |
| PCK2-F | 5’- GGCTGAGAATACTGCCACACT |
| PCK2-R | 5’- ACCGTCTTGCTCTCTACTCGT |
| qPCR primers for testing homo sapiens miRNA | |
| miR-211-F | 5’- CCTTTGTCATCCTTCGC |
| miR-211-R | 5’- GAACATGTCTGCGTATCTC |
| miR-145-F | 5’- GTCCAGTTTTCCCAGGA |
| miR-145-R | 5’- GAACATGTCTGCGTATCTC |
| miR-143-F | 5’- GCAGTGCTGCATCTCTG |
| miR-143-R | 5’- GAACATGTCTGCGTATCTC |
| miR-204-F | 5’- CCTTTGTCATCCTATGCC |
| miR-204-R | 5’- GAACATGTCTGCGTATCTC |
| miR-9-F | 5’- TCTTTGGTTATCTAGCTGT |
| miR-9-R | 5’- GAACATGTCTGCGTATCTC |
| miR-449b-F | 5’- GCAGTGTATTGTTAGCTG |
| miR-449b-R | 5’- GAACATGTCTGCGTATCTC |
| qPCR primers for testing Ch-IP assay | |
| FBP1-R1-F | 5’- CTAGCTCTCCCCTAGTAACT |
| FBP1-R1-R | 5’- AGGCTGAGGCGGGCAGATCT |
| FBP1-R2-F | 5’- AGATCTGCCCGCCTCAGCCT |
| FBP1-R2-R | 5’- ACCTGTAATCCCAGCACTTC |
| FBP1-R3-F | 5’- GAAGTGCTGGGATTACAGGT |
| FBP1-R3-R | 5’- GAAAATTGACTGCTGGATTC |
| FBP1-R4-F | 5’- GAATCCAGCAGTCAATTTTC |
| FBP1-R4-R | 5’- TGGGGAACACCGAGAGGGGC |
| FBP1-R5-F | 5’- GCCCCTCTCGGTGTTCCCCA |
| FBP1-R5-R | 5’- TGACTTTGACTAGGAGGAAC |
| FBP1-R6-F | 5’- GTTCCTCCTAGTCAAAGTCA |
| FBP1-R6-R | 5’- TAGAAAAGGAAGACACGGAA |
| FBP1-R7-F | 5’- TTCCGTGTCTTCCTTTTCTA |
| FBP1-R7-R | 5’- CGGTCGCTGACACAGAGTCC |
| FBP1-R8-F | 5’- GGACTCTGTGTCAGCGACCG |
| FBP1-R8-R: | 5’- CACCAGCTAGGCAGCGAAAC |
| FBP1-R9-F | 5’- GTTTCGCTGCCTAGCTGGTG |
| FBP1-R9-R | 5’-CCCGCCCCCGGGAACACTC |
| FBP1-R10-F | 5’-GAGTGTTCCCGGGGGCGGG |
| FBP1-R10-R | 5’-TGTTGACGTCCGTGTCGAAG |
| PCK2-R1-F | 5’- ATGTGGGGGTGACAACATTA |
| PCK2-R1-R | 5’- TGAAATGTTCTAATTGTTTAC |
| PCK2-R2-F | 5’- GTAAACAATTAGAACATTTCA |
| PCK2-R2-R | 5’- TGTTTCACAACTATATTTTAC |
| PCK2-R3-F | 5’- GTAAAATATAGTTGTGAAACA |
| PCK2-R3-R | 5’- TCTTATCCTGCATAGCTAATC |
| PCK2-R4-F | 5’- GATTAGCTATGCAGGATAAGA |
| PCK2-R4-R | 5’- GTCCCCGTCTCTTCCCCCTC |
| PCK2-R5-F | 5’- GAGGGGGAAGAGACGGGGAC |
| PCK2-R5-R | 5’- GCTGAGTTGCTGAGCTGGTA |
| PCK2-R6-F | 5’- TACCAGCTCAGCAACTCAGC |
| PCK2-R6-R | 5’- GACCACTGGATATCACCCAC |
| PCK2-R7-F | 5’- GTGGGTGATATCCAGTGGTC |
| PCK2-R7-R | 5’- TGCAGAGTAGCTGGAATTAC |
| PCK2-R8-F | 5’- GTAATTCCAGCTACTCTGCA |
| PCK2-R8-R | 5’- CCTGGGCCTTTCTGTAGTCC |
| PCK2-R9-F | 5’- GGACTACAGAAAGGCCCAGG |
| PCK2-R9-R | 5’- GCCCAGCCGCCGCCCCAACC |
| PCK2-R10-F | 5’- GGTTGGGGCGGCGGCTGGGC |
| PCK2-R10-R | 5’- TGGGCCCCGGGCCGGGGGTC |

| oligonucleotides | |
| --- | --- |
| pGIPZ control oligonucleotide | 5’-CTCGCTTGGGCGAGAGTAA-3’ |
| pGIPZ-AF9-shRNA-1 | 5’-ACTTGCTCATGTCTGTTCA-3’ |
| pGIPZ-AF9-shRNA-2 | 5’-GCACAGTAACATACAGCACTT-3’ |
| FBP1-R | 5’- TTCTTCTGACACGAGAACACAC |
| PCK2-F | 5’- GGCTGAGAATACTGCCACACT |
| PCK2-R | 5’- ACCGTCTTGCTCTCTACTCGT |
